# Competitive interactions in two different plant species: Do grassland mycorrhizal communities and nitrogen addition play the same game?

**DOI:** 10.3389/fpls.2023.1084218

**Published:** 2023-03-13

**Authors:** Ali Bahadur, Shengjing Jiang, Wei Zhang, Wasim Sajjad, Muhammad Usman, Fahad Nasir, Muhammad Amir Zia, Qi Zhang, Jianbin Pan, Yongjun Liu, Tuo Chen, Huyuan Feng

**Affiliations:** ^1^ Key Laboratory of Extreme Environmental Microbial Resources and Engineering, Northwest Institute of Eco-Environment and Resources, Chinese Academy of Sciences, Lanzhou, China; ^2^ MOE Key Laboratory of Cell Activities and Stress Adaptation, School of Life Sciences, Lanzhou University, Lanzhou, China; ^3^ State Key Laboratory of Cryospheric Science, Northwest Institute of Eco-Environment and Resources, Chinese Academy of Sciences, Lanzhou, China; ^4^ State Key Laboratory of Herbage Improvement and Grassland Agro-ecosystems, College of Pastoral Agriculture Science and Technology, Lanzhou University, Lanzhou, China; ^5^ State Key Laboratory of Grassland Agroecosystems, Key Laboratory of Grassland Livestock Industry Innovation, Ministry of Agriculture and Rural Affairs, College of Pastoral Agriculture Science and Technology, Lanzhou University, Lanzhou, Gansu, China; ^6^ Key Laboratory of Mollisols Agroecology, Northeast Institute of Geography and Agroecology, Chinese Academy of Sciences, Changchun, Jilin, China; ^7^ National Institute for Genomics and Advanced Biotechnology, National Agriculture Research Center, Islamabad, Pakistan

**Keywords:** host plant, grassland AMF inoculum, nitrogen deposition, plant competition, non-host plant

## Abstract

In the Tibetan Plateau grassland ecosystems, nitrogen (N) availability is rising dramatically; however, the influence of higher N on the arbuscular mycorrhizal fungi (AMF) might impact on plant competitive interactions. Therefore, understanding the part played by AMF in the competition between *Vicia faba* and *Brassica napus* and its dependence on the N-addition status is necessary. To address this, a glasshouse experiment was conducted to examine whether the grassland AMF community’s inocula (AMF and NAMF) and N-addition levels (N-0 and N-15) alter plant competition between *V. faba* and *B. napus*. Two harvests took day 45 (1^st^ harvest) and day 90 (2^nd^ harvest), respectively. The findings showed that compared to *B. napus*, AMF inoculation significantly improved the competitive potential of the *V. faba*. In the occurrence of AMF, *V. faba* was the strongest competitor being facilitated by *B. napus* in both harvests. While under N-15, AMF significantly enhanced tissue N:P ratio in *B. napus* mixed-culture at 1^st^ harvest, the opposite trend was observed in 2^nd^ harvest. The mycorrhizal growth dependency slightly negatively affected mixed-culture compared to monoculture under both N-addition treatments. The aggressivity index of AMF plants was higher than NAMF plants with both N-addition and harvests. Our observation highlights that mycorrhizal associations might facilitate host plant species in mixed-culture with non-host plant species. Additionally, interacting with N-addition, AMF could impact the competitive ability of the host plant not only directly but also indirectly, thereby changing the growth and nutrient uptake of competing plant species.

## 1 Introduction

The amount of biologically available N is dramatically rising in various ecosystems worldwide because of anthropogenic activities (Jiang et al., 2018). Worldwide application of N may have caused significant changes in aboveground plant diversity and production and the composition and diversity of underground microbial community (Zhou et al., 2016; Qin et al., 2022). The efficacy of grassland may be affected by competitions between legume and non-legume plants, due to the changes in availability of macronutrients, particularly phosphorus (P) and N. During plant competition, plant biomass and abundance are inhibited by N enrichment, and P is usually more effective in enhancing the competitiveness of legume plants than N addition (Unger et al., 2021). Furthermore, N-addition may weaken plant-microbe interactions, induce dormancy, and causes indigenous destruction of vulnerable functional grouping of microbes (Kearns et al., 2016; Jiang et al., 2018). In this study, we investigated the interspecific interactions under N-addition and their influence on the colonization of arbuscular mycorrhizal fungi (AMF).

Among the widespread root-associated microbes, AMF are known to be sensitive to N-addition in both pot and field experiments. Interaction between AMF and plants generally plant supply related AMF with carbohydrates, and in return, fungi provide soil P, and possible N to their host plants (Liu et al., 2015; Bahadur et al., 2019b). The mycorrhizal symbiotic association with plant roots may also improve plants ability to protect themselves against several types of biotic and abiotic stresses and root pathogens (Lewandowski et al., 2013; Zhou et al., 2022; Bahadur et al., 2019a). Recently, numerous studies have reported that N-addition will reduce AMF community diversity and abundance (Chen et al., 2017; Williams et al., 2017); nonetheless, positive or even neutral effects of N-addition have also been reported in various studies (Zheng et al., 2014; Unger et al., 2021). For instance, a field experiment conducted in Qinghai-Tibet Plateau reports that AMF spore density is not affected by N-addition, while the extraradical hyphal density dramatically increases (Zheng et al., 2016). Such different opinions and observations propose that AMF performance fluctuates under N-addition and hence necessitates further studies.

Knowledge of plant-fungal processes and mycorrhizal functioning are crucial for the competitive interactions between the host and non-host plant species. For instance, *Vicia faba* and *Brassica napus* are the main competitive interactions adopted by farmers in China. These competitive interactors respond differently to AMF, since they have different nutrient acquisition strategies and nutrient demands (Meng et al., 2015; Qiao et al., 2015). N captured by non-host plant *B. napus* may favor the occurrence of AMF, when *B. napus* is inter-cropped with the host plant *Plantago lanceolate* (Hodge, 2003). They are similar in understanding the functioning of local mycorrhizal effects on *B. napus* under N-addition (Carneiro et al., 2015; Pakpour and Klironomos, 2015). The presence of AMF in and *Zea mays* growth may both be adversely affected by *B. napus* (De Souza and Santos, 2018). Previous studies report that competitive interspecific interactions could be altered and regulated by AMF complete reallocating nutrients as reported in numerous host plants (Fellbaum et al., 2014; Bever, 2015), for example the legume and grass, the nutritional competition of legumes is encouraged the presence of AM fungus (Scheublin et al., 2007; Wagg et al., 2011). When grown with *Hordeum vulgare*, *V. faba* demonstrates a higher ability to compete nutrients mediated by AMF (Bahadur et al., 2019b). AMF may alter competitive associations between host and non-host plant species by favoring the mycorrhizal plants over the neighboring ones, generally due to the positive mycorrhizal responsiveness.

So far, studies investigating the impacts of AMF on nutrient competition usually focus on the host plants only (Ren et al., 2017; Yang et al., 2018; Bahadur et al., 2019b), it is still not clear how AMF affect the nutrient competition between the host and non-host plant species when they are inter-cropped. Moreover, previous meta-analysis showed that nitrogen addition reduced plant species-diversity, but increase plant productivity (Fu and Zhen-Xi, 2016). Other field warming studies (e.g., Fu et al., 2019; Wang et al., 2021; Zhang et al., 2021) indicated that plant productivity increased with soil nitrogen availability. However, whether and how N deposition regulates AMF affecting the plant nutrient competition is largely unknown. Here, we investigate that under different N-addition levels: (1) how AMF inoculation facilitates the competitive ability and growth performance of host (i.e., *V. faba*) and non-host (i.e., *B. napus*) species, and (2) whether the AMF community contributes differently to the plant competitive interactions. We tested the hypothesis that both AMF inoculation and N-addition could promote plant competitive ability. Our results provide essential information to develop strategies for crop management.

## 2 Materials and methods

### 2.1 Sampling region description

This study was conducted in a glasshouse. The soil used for this experiment was collected from the Alpine Meadow and Wetland Ecosystem Research Station (Aziz Station) (33°40′N, 101°51′E; c. 3,500 m a. s. l.; 5° slope), Lanzhou University, Gannan Tibet Autonomous Prefecture, Gansu Province, China. The area of soil collection has been fenced and grazed from November to April since 2011. The soil type is classified as Cambisol (FAO taxonomy), with a pH of 8.5 and the available P of 19.68 mg in 1 kg soil. The mean annual temperature and precipitation are 2.2°C and 672 mm, respectively. N-addition of approximately 14.26-18.65 kg ha^-1^ yr^-1^ was applied in this area. Therefore, soil N concentrations gradually increased, resulting in the shift of plant community towards dominance by *Elymus nutans* and the decrease of plant species richness (Liu et al., 2015).

### 2.2 Experiment design

The experiment was carried out from October 8, 2018, to January 15, 2019, in a glasshouse at 16 to 23C, and 25% relative humidity with a photoperiod of 14 h light and 10 h dark and during the plant growth period. The mycorrhizal inocula were propagated in a 1:1 (sand/soil = v/v) mixture of washed river sand and soil (< 2 mm thickness), which included fine root fragments, mycelia, and around 60 spores per gram. The soil was sterilized at 121°C for two hours for two consecutive days after being sieved through a 2-mm sieve.

**Figure 1:**
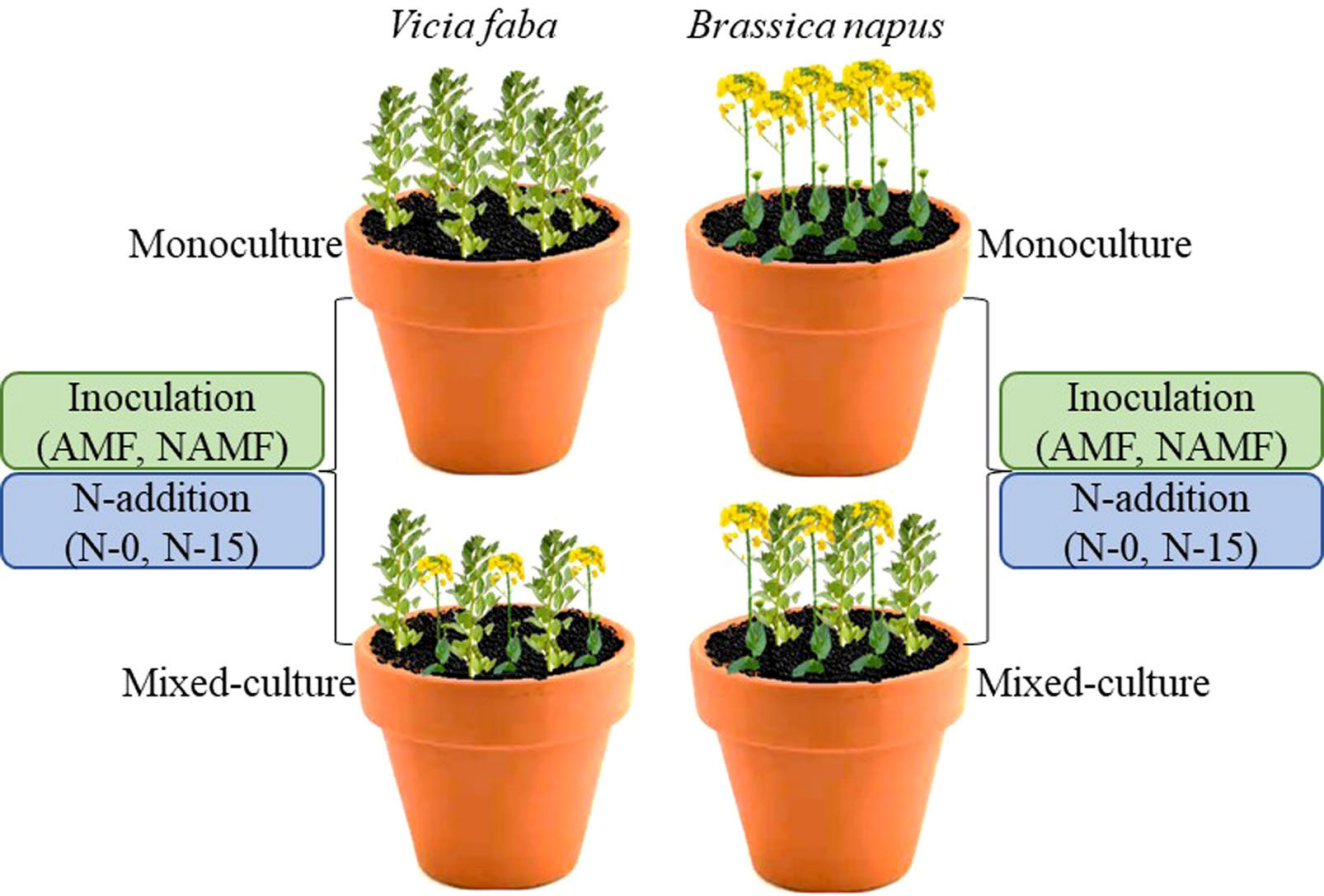
In monoculture and mixed-culture *V. faba* and (*B*) *napus* under AMF inoculation and N-addition conditions of N-0 and N-15 were imposed for both harvests (Day 45 1^st^ harvest and Day 90 2^nd^ harvest).

To prepare AMF inoculation for experiment, we deposited 1.4 kg of soil into plastic pots (10 cm in length, 11 cm in width, and 12 cm in height) after a sponge was placed on the bottom, and then evenly spread 60 g inocula (≈ 2000 spores per pot) on the middle and 400 g soil on the top. To minimize changes in nutrient availability and microbial communities between AMF and NAMF treatments, each non-mycorrhizal pot received 5 mL of AMF-free soil filtrate (Bahadur et al., 2019b). To measure AMF root colonization and its effects on plant nutrient competition, we carried a pot experiment with a factorial randomized complete design: AMF inoculation (AMF and NAMF, with and without mycorrhizal inocula) × N-addition (N-0 and N-15, 0 and 15 g N m^-2^ yr^-1^, respectively) × plant culture type (monoculture and mixed-culture) × plant species (*V. faba* and *B. napus*). There were eight replicates for each of the 16 combination treatments, resulting in 128 pots in total. For the monoculture, six plants *V. faba* or *B. napus* were planted per pot; and for the mixed-culture, there were three *V. faba* and three *B. napus* plants per pot. The pots were rotated randomly after every seven days. During the experiment, pots were watered 2-3 times a week with 100 mL water each time. The modified Hoagland solution (20 mL mixture of 25% ferric salt and 12.5% phosphate) was added to provide nutrients (Hoagland and Arnon, 1950) at the 2-week interval after sowing. Seeds *B. napus* and *V. faba* were provided by the GPIA (Gannan Prefecture Institute of Agronomy), China. Seeds were surface-sterilized (at 70% of ethanol for 1 min, rinsed with 0.5% NaClO solution for 2 mins for *B. napus* and 5 mins for *V. faba*, and 32% of HCl one drop for 5 mins) and soaked in warm water (50°C for 20 mins for *B. napus* and 55°C 10 mins for *V. faba*). Soil collected from the grazing area contained N-addition (NH_4_NO_3_) was used for experiment each year. Plants were harvested twice, one on day 45 and another on day 90 after sowing, and the above- and below-ground materials were collected and separated (**Figure 1**). The roots were cleaned using tap water and rinsing in distal water. In order to measure AMF colonization, root subsamples were stored at 4°C. The root and shoot biomass were recorded after oven drying at 80°C for 48 hr.

### 2.3 AMF root colonization

To measure the mycorrhizal colonization, the roots of AMF plants were cut into small fragments and washed to remove any remaining soil. The root fragments were randomly collected and washed with distilled water after being bleached for 25 mins in a water bath containing 10% KOH at 80°C. After that staining with 0.05% (v/v) trypan blue in lactic acid at 80°C for 25 mins (Phillips and Hayman, 1970). The root fragments were placed on slides to determine the AMF colonization in roots under a 200 × compound microscope.

### 2.4 Tissue nitrogen and phosphorus content

To measure the P and N contents, dried aboveground plant materials were ground and sieved. Further, 2-5 mg of powdered plant material was moved and then digested in 5 mL 98% with H_2_SO_4_ solution, adding 1 g mixture of catalyst K_2_SO_4_ and CuSO_4_ (10: 1 w/w) in a 375°C -digestion furnace. The extract was then filtered using Smartchem 200 (AMS, Italy). The molybdovanado-phosphate technique was used to colorimetrically measure the plant P content (Lü and Tian, 2007), and the micro-Kjeldahl technique was used to quantify N content (Mckenzie and Wallace, 1954).

### 2.5 Statistical analysis

We evaluated the mycorrhizal response in terms of plant N and P contents and plant biomass to calculate the changes in AMF colonization under different N-addition levels. Mycorrhizal growth dependency (MGD) of plants (monoculture or mixture plant type) was calculated according to the following formula (Smith and Read, 2008): MGD = 100 × (Biomass_AMF_ Biomass_NAMF_)/Biomass_AMF_; Where Biomass_AMF_ and Biomass_NAMF_ represented the average of the total biomass of mycorrhizal and non-mycorrhizal plants, respectively. A negative and positive values for MGD indicates that AMF suppresses and promotes plant growth, respectively.

In addition, the competitive capability of *V. faba* and *B. napus* in a plant mixture was measured using the aggressivity index (AGR) and relative interaction intensity index (RII), both of which were calculated using the total biomass of plant species. The AGR of plant *i* relative to plant *j* was calculated according to Mcgilchrist and Trenbath (1971): AGR*_ij_* = RY*_i_* RY*_j_* = (DM*_ij_*/DM*_ii_*) (DM*_ji_*/DM*_jj_*), where RY*i* and RY*j* are the relative yield of plants *i* and *j*, respectively; DM*ii* and DM*jj* are the dry biomass of monoculture plants *i* and *j*, respectively; and DM*ij* and DM*ij* are the dry biomass of mixed-culture plants *i* and *j*, respectively. If the AGR*ij* value is zero, then *i* and *j* plants have a similar competitive capability. An AGR*ij* value > 0 showes a strong competitive capability of plant *i* compared to plant *j*, and an AGR*ij* value < 0 indicates a weaker competitive capability of plant *i* comapre to plant *j*. Furthermore, the RII evaluated a simple comparison of competition strength across treatments and species according to Armas et al. (2004): RII = (DM*_ij_* DM*_ii_*)/(DM*_ij_* + DM*_ii_*). The RII is an evaluated of the strength of competition between species and is centered on zero with negative interactions (competition) designated by values between 0 and −1, and positive interactions (facilitation) indicated by values between 0 and +1.

IBM SPSS version 19.0 for Windows (SPSS Inc., Chicago, USA) was used for statistical analyses. To examine the impacts of N-addition and AMF inoculation on the AGR and RII, a two-way ANOVA was used. Data on the total biomass, root:shoot ratio, plant N and P content, and N:P were analyzed using a mixed linear model with N-addition and mycorrhization. A Duncan’s multiple range test was used for multiple comparisons.

## 3 Results

### 3.1 AMF root colonization

Roots of B. napus and V. faba in the NAMF treatment were not colonized by AMF; nevertheless, a high level of AMF root colonization was detected in both plant species for the 1^st^ and 2^nd^ harvests (**Table 1**). At the 1^st^ harvest, root colonization in the monoculture V. faba was significantly higher than that in the monoculture B. napus in N-0 (P < 0.05) with no significant difference detected between the mixed-culture V. faba and B. napus; in N-15, both monoculture and mixed-culture V. faba had significantly higher AMF root colonization rate compared to that of B. napus (P < 0.05) (**Table 1**). At the 2^nd^ harvest, a significantly higher AMF root colonization rate was detected in mixed-culture V. faba with a significantly lower rate found in monoculture B. napus, regardless of N-addition levels (P < 0.05) (**Table 1**).

**Table 1:** Proportion of mycorrhizal colonization (mean ± SE) in monoculture and mixed-culture *V. faba* and *B. napus* under AMF inoculation and N-addition conditions of N-0 and N-15.

1^st^ harvest (Day 45)			2^nd^ harvest (Day 90)	
Plants	N-0	N-15	N-0	N-15
Monoculture *V. faba*	34.8 ± 3.1a	39.5 ± 3.8a	22.5 ± 1.9b	14.3 ± 1.7bc
Mixed-culture *V. faba*	31.2 ± 3.0a	41.1 ± 6.2a	55.6 ± 3.6a	48.8 ± 5.3a
Monoculture *B. napus*	3.3 ± 1.5b	12.1 ± 2.4b	4.1 ± 0.4c	6.4 ± 1.2c
Mixed-culture *B. napus*	30.8 ± 3.8a	11.1 ± 2.3b	28.3 ± 5.0b	23.0 ± 2.2b
*df*	3	3	3	3
*F*	23.96	17.28	43.32	36.24
*P*	**0.000**	**0.000**	**0.000**	**0.000**

Means with the same letter in each column are not significantly different (Duncan test: *P* < 0.05). Significant effects of treatments are indicated in bold.

### 3.2 Plant growth performance

In the 1^st^ harvest, the total biomass of V. faba was significantly affected by culture type only (P < 0.05), with no significant difference detected for the interactions between culture type, inoculation, and N-addition (**Table 2**). The total biomass of B. napus was significantly affected by culture type and inoculation, while no significant effect was observed on N-addition; however, there were significant interactions found between culture type and inoculum (C × I), inoculation and N-addition (I × N), and among the three factors (C × I × N) (P < 0.05) (**Table 2**). In the 2^nd^ harvest, treatments and interactions significantly affected the total biomass of V. faba (P < 0.05) except the interaction of C × N; the N-addition and the interaction of C × I did not significantly affect the total biomass of B. napus (**Table 2**).

**Table 2:** Analyses of variance (ANOVA) for total biomass, root:shoot ratio, tissue N and P content, and N:P ratio of culture type *V. faba* and *B. napus* under AMF inoculation and N-addition conditions of N-0 and N-15.

	Total biomass			R:S ratio		Tissue N content		Tissue P content		Tissue N:P ratio	
	*df*	*F*	*P*	*F*	*P*	*F*	*P*	*F*	*P*	*F*	*P*
1^st^ harvest (Day 45)											
*V. faba*											
Culture type (C)	1	89.62	**0.000**	2.64	0.117	27.08	**0.000**	15.39	**0.001**	5.03	**0.034**
Inoculation (I)	1	0.72	0.406	0.04	0.844	0.34	0.567	2.22	0.149	0.68	0.416
N-addition (N)	1	3.11	0.091	0.27	0.605	0.26	0.616	0.53	0.475	11.78	**0.002**
*C × I*	1	2.60	0.120	0.28	0.602	0.24	0.632	2.04	0.167	0.10	0.752
*C × N*	1	1.07	0.311	0.02	0.878	2.37	0.137	2.47	0.129	0.05	0.831
*I × N*	1	0.27	0.611	2.17	0.154	1.37	0.253	2.41	0.134	0.29	0.594
*C × I × N*	1	0.03	0.859	0.42	0.524	0.84	0.369	0.64	0.430	1.30	0.266
*B. napus*											
Culture type (C)	1	47.79	**0.000**	4.34	**0.048**	32.46	**0.000**	34.99	**0.000**	0.18	0.677
Inoculation (I)	1	16.41	**0.000**	0.19	0.661	5.57	**0.027**	8.26	**0.008**	0.04	0.849
N-addition (N)	1	1.33	0.261	1.41	0.246	0.40	0.533	1.30	0.266	4.22	**0.051**
*C × I*	1	5.43	**0.028**	0.47	0.498	1.11	0.302	3.74	0.065	5.35	**0.030**
*C × N*	1	2.12	0.158	1.88	0.183	0.92	0.348	2.05	0.165	0.10	0.756
*I × N*	1	12.61	**0.002**	0.00	0.995	12.63	**0.002**	11.41	**0.002**	0.34	0.566
*C × I × N*	1	12.45	**0.002**	0.08	0.780	11.61	**0.002**	11.92	**0.002**	0.19	0.670
2^nd^ harvest (Day 90)											
*V. faba*											
Culture type (C)	1	424.03	**0.000**	1.88	0.183	50.90	**0.000**	65.14	**0.000**	0.74	0.397
Inoculation (I)	1	82.05	**0.000**	6.18	**0.020**	41.61	**0.000**	0.81	0.377	46.63	**0.000**
N-addition (N)	1	8.67	**0.007**	7.37	**0.012**	3.88	**0.051**	9.00	**0.006**	25.87	**0.000**
*C × I*	1	11.17	**0.003**	0.39	0.540	1.51	0.230	2.43	0.132	0.11	0.742
*C × N*	1	0.12	0.735	0.47	0.540	2.88	0.103	0.90	0.360	3.47	0.075
*I × N*	1	5.02	**0.035**	1.94	0.176	9.69	**0.005**	1.12	0.301	17.82	**0.000**
*C × I × N*	1	3.89	**0.050**	1.07	0.311	6.58	**0.017**	8.36	**0.008**	0.00	0.959
*B. napus*											
Culture type (C)	1	119.52	**0.000**	0.04	0.839	50.90	**0.000**	77.20	**0.000**	28.32	**0.000**
Inoculation (I)	1	15.10	**0.001**	5.87	**0.023**	41.61	**0.000**	9.73	**0.005**	8.76	**0.007**
N-addition (N)	1	0.34	0.564	0.85	0.365	3.88	**0.061**	0.53	0.474	24.30	**0.000**
*C × I*	1	0.56	0.461	0.00	0.995	1.51	0.230	2.36	0.138	5.30	**0.030**
*C × N*	1	13.39	**0.001**	9.79	**0.005**	2.88	0.103	8.45	**0.008**	16.97	**0.000**
*I × N*	1	28.77	**0.000**	0.18	0.677	9.69	**0.005**	39.43	**0.000**	1.94	0.177
*C × I × N*	1	56.06	**0.000**	0.94	0.343	6.58	**0.017**	52.46	**0.000**	4.13	**0.053**

Significant effects of treatments are indicated in bold.

**Figure 2:**
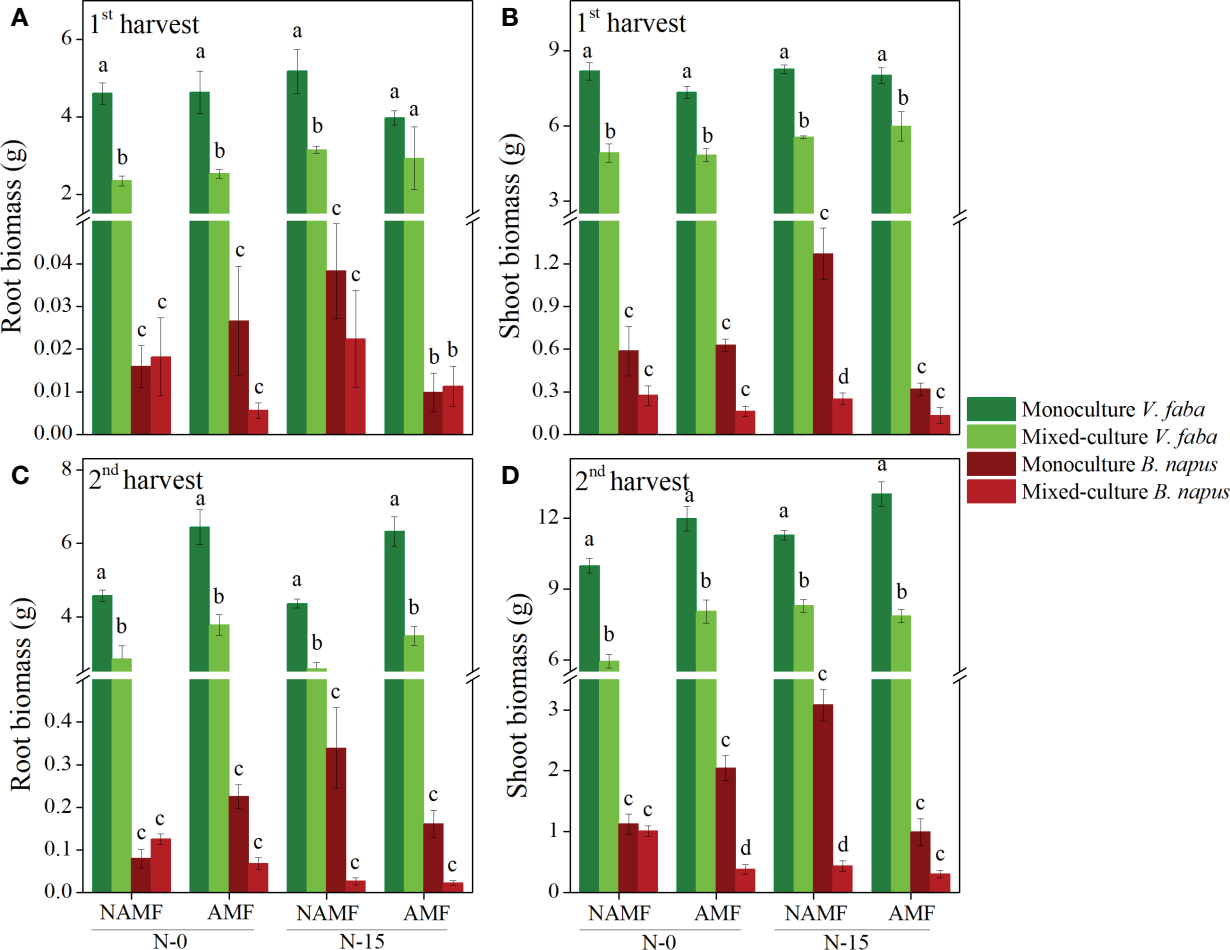
Root and shoot biomass production per plant (mean ± SE) in monoculture and mixed-culture *V. faba* and *B. napus* under AMF inoculation and N-addition conditions of N-0 and N-15. **(A, B)** show 1st harvest, **(C, D)** show 2nd harvest. Different letters denote significantly different means (Duncan test: *P* < 0.05).

The shoot and root biomass of V. faba was significantly higher than that of B. napus regardless of culture type, inoculation, and N-addition, with a significant decrease found in mixed-culture V. faba in both harvests, except for the root biomass under conditions of AMF inoculation and N-15 in the 1^st^ harvest (P < 0.05) (**Figure 2**). Compared to the mixed-culture B. napus, the monoculture B. napus had significantly greater shoot biomass at NAMF+N-15 in the 1^st^ and 2^nd^ harvests and at AMF+N-0 in the first harvest (P < 0.05) (**Figure 2**).

In the 1^st^ harvest, treatments had no significant effect on root:shoot ratio in V. faba, whereas only culture type significantly affect B. napus root:shoot ratio (P < 0.05) (**Table 2**). In the 2^nd^ harvest, the root:shoot ratio of V. faba was significantly affected by inoculation and N addition, whereas the root:shoot ratio of B. napus was significantly influenced by inoculation and the interaction of C × N (**Table 2**).

### 3.3 Tissue nutrients

The culture type significantly affected N and P contents in *V. faba*, and culture type, inoculation, and interactions *I* × *N* and *C* × *I* × *N* significantly affected the N and P contents in *B. napus* (*P* < 0.05) (**Table 2**). Culture type, inoculation, and interactions *I* × *N* and *C* × *I* × *N* significantly affected the N contents in *V. faba* and *B. napus* in the 2^nd^ harvest. The P contents in *V. faba* was significantly affected by culture type, N-addition and interactions C × I × N, whereas culture type, inoculation, and interactions *C* × *N*, *I* × *N*, and *C* × *I* × *N* significantly affected the P contents in *B. napus* (*P* < 0.05) (**Table 2**). In addition, in the 1^st^ harvest, the N:P ratio in *V. faba* was significantly affected by culture type and N-addition, while N:P ratio in *B. napus* was significantly affected by N-addition and the interaction of *C* × *I* (*P* < 0.05) (**Table 2**). In the 2^nd^ harvest, the N:P ratio in *V. faba* was significantly affected by inoculation, N-addition, and interactions *I* × *N*, while treatments and interactions significantly affected the N:P ratio in *B. napus* (*P* < 0.05) except the interaction of *I* × *N* (**Table 2**).

**Figure 3:**
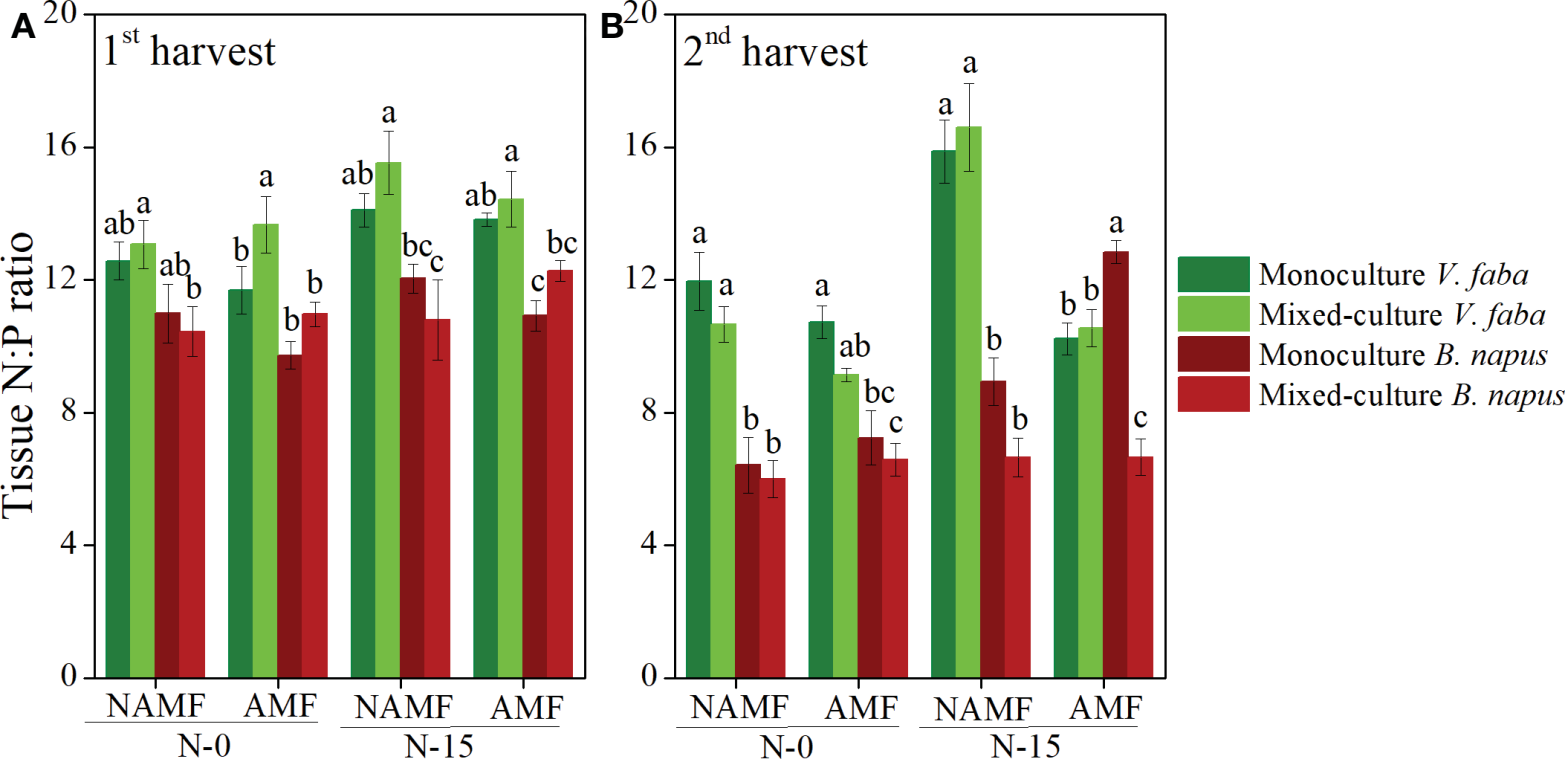
Tissue N:P ratio per plant (mean ± SE) in monoculture and mixed-culture *V. faba* and *B. napus* under AMF inoculation and N-addition conditions of N-0 and N-15. **(A)** shows 1st harvest, **(B)** shows 2nd harvest. Different letters denote significantly different means (Duncan test: *P* < 0.05). For statistical details, see **Table 2** and **Supplementary Table 1**.

The N:P ratio of V. faba was higher than that of B. napus regardless of culture type, inoculation, and N-addition in both harvests, except for the N:P ratio under condition of AMF inoculation and N-15 in the 2^nd^ harvest (*P* < 0.05) (**Figure 3**). Compared to the monoculture B. napus, the mixed-culture B. napus had higher N:P ratio at AMF+N-15 and AMF+N-0 in the1^st^ harvest, while compared to mixed-culture B. napus, monoculture B. napus had higher N:P ratio in the 2^nd^ harvest (**Figure 3**).

### 3.4 Mycorrhizal growth dependency

**Figure 4:**
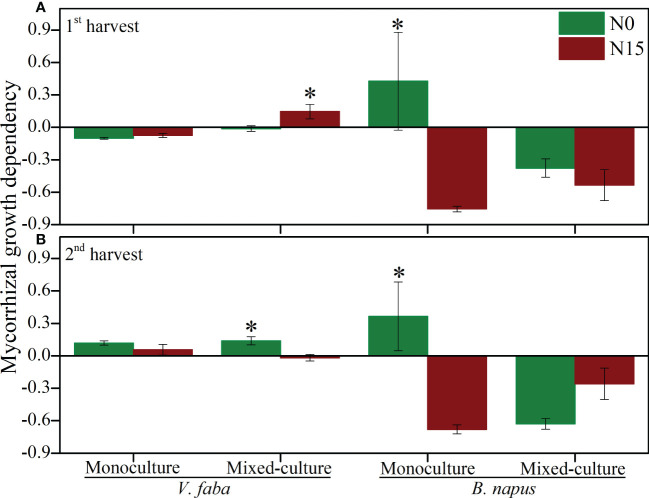
Mycorrhizal growth dependency (MGD) of *V. faba* and *B. napus* under N-addition conditions of N-0 and N-15. **(A)** shows 1st harvest, **(B)** shows 2nd harvest. * denotes significant difference.

The mycorrhizal growth dependency (MGD) was varied in cultural type and N-addition in both harvests (**Figure 4**). The MGD was negatively affected by N-0 and N-15 in 1^st^ harvest and positively affected in 2^nd^ harvest in monoculture V. faba. However, MGD was positively affected by N-15 in 1^st^ harvest, while negatively affected by N-15 in 2^nd^ harvest in mixed-culture V. faba. The MGD of monoculture and mixed-culture B. napus was negatively affected by N-addition in both harvests, except for the N-0 under monoculture B. napus in both harvest (**Figure 4**).

### 3.5 Competitiveness

**Figure 5:**
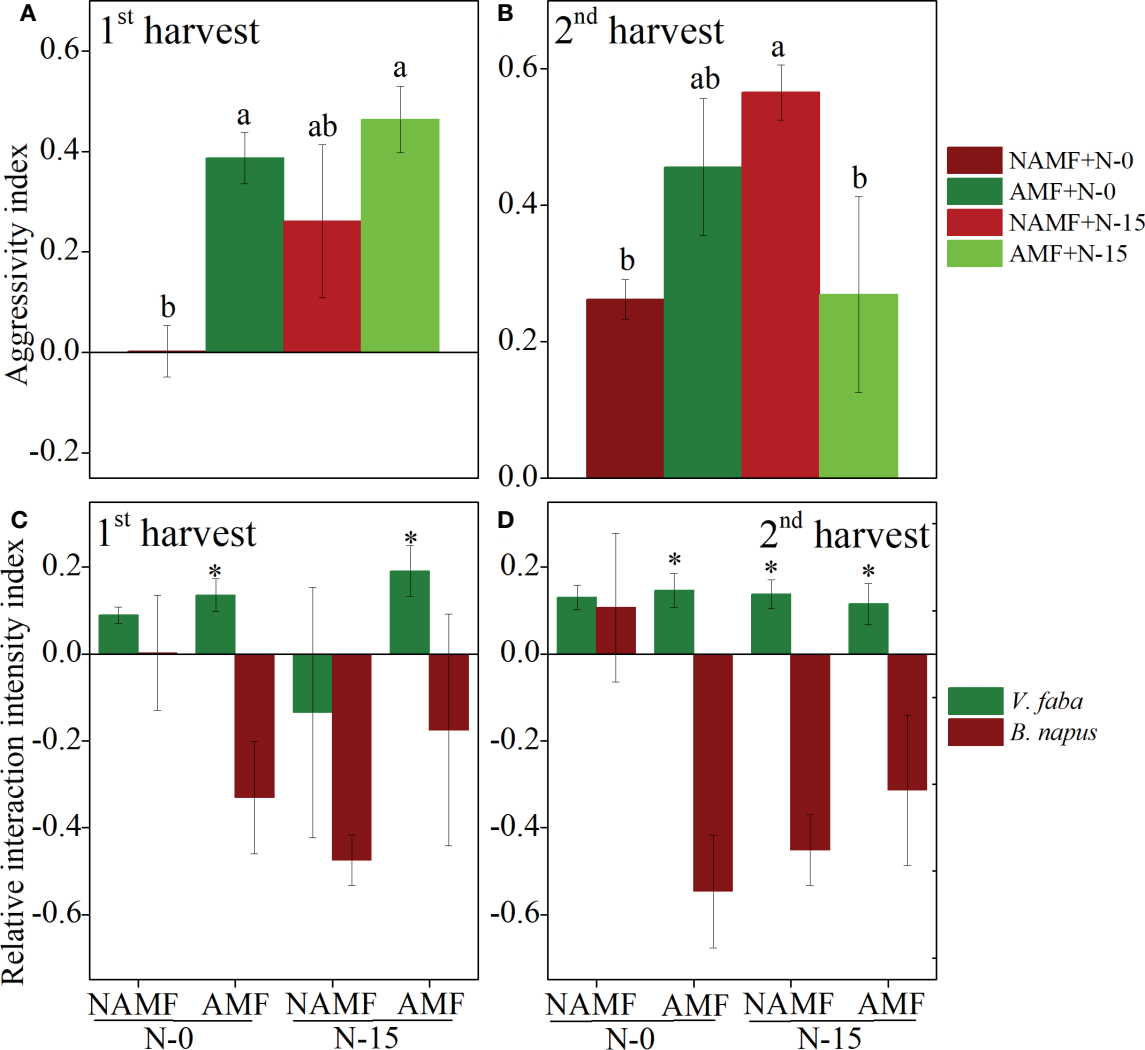
Effects of AMF inoculation on *V. faba* and *B. napus* nder N-addition conditions of N-0 and N-15, evaluated using the aggressivity index (AGR) and relative interaction intensity index (RII). **(A, C)** show 1st harvest, **(B, D)** show 2nd harvest. For statistical details, see **Table 3**. * denotes significant difference.

The aggressivity index (AGR) of AMF plants was comparatively higher than that of NMAF plants, with a significant decrease found in NAMF+N-0 in the 1^st^ harvest, indicating a more remarkable competitive ability of AMF plants (**Figure 5A**). The AGR of AMF plants was significantly higher than that of NAMF plants at N-0, while AMF plants was significantly lower than that of NAMF at N-15 in the 2^nd^ harvest (**Figure 5B**). In addition, according to the analysis of the relative interaction intensity index (RII), in the 1^st^ harvest, the growth of B. napus was suppressed by V. faba when inoculated with AMF+N-0. Compared to the NAMF+N-15, the B. napus facilitated the growth of AMF+N-15 (RII > 0) V. faba plants (**Figure 5C**). In the 2^nd^ harvest, plant growth of B. napus was suppressed by V. faba at AMF+N-0, competitive suppression existence highest (RII < 0). Unlike the effects of AMF, when B. napus were at NAMF+N-0, the plant growth was facilitated by V. faba (RII < 0) (**Figure 5D**).

In the 1^st^ harvest, the results showed that AGR was significantly affected by the N-addition, inoculation and interaction *N × I* (P < 0.05), while RII of V. faba and B. napus was not significantly affected by treatments and interactions (**Table 3**). In the 2^nd^ harvest, the AGR was significantly affected by interactions *N × I* (P < 0.05), whereas no significant effect was observed on inoculation and N-addition. The RII of V. faba and B. napus was not significantly affected by inoculation, N-addition, and interactions, except for the B. napus under interactions *N × I* (P < 0.05) (**Table 3**).

**Table 3:** Effects of AMF inoculation and N-addition on the aggressivity index (AGR) and relative interaction intensity indices (RII) for *V. faba* and *B. napus*.

1^st^ harvest (Day 45)								2^nd^ harvest (Day 90)					
	AGR			*V. faba* RII		*B. napus* RII		AGR		*V. faba* RII		*B. napus* RII	
	*df*	*F*	*P*	*F*	*P*	*F*	*P*	*F*	*P*	*F*	*P*	*F*	*P*
N-addition (N)	1	14.20	**0.003**	0.83	0.381	0.84	0.380	0.55	0.473	0.09	0.773	00.67	0.430
Inoculation (I)	2	17.35	**0.000**	1.45	0.276	0.061	0.941	0.26	0.777	0.02	0.981	2.52	0.126
*N × I*	1	6.56	**0.026**	1.64	0.227	3.18	0.102	7.04	**0.022**	0.22	0.650	5.61	**0.037**

Significant effects of treatments are indicated in bold.

## 4 Discussion

As described by Meng et al. (2015) and Qiao et al. (2015), farmers in China mainly used *V. faba* and *B. napus* as competitive interactors. However, little is known about the influence of AMF communities and N-addition on this competitive interaction. Most previous studies (both field and glasshouse studies) have also reported that N enrichment, mainly N-addition at high levels, reduces AMF functions and shifts the mycorrhizal symbiosis toward parasitism (Liu et al., 2015; Jiang et al., 2018).

The current results demonstrate that AMF in mixed-culture are directly essential for the survival of host plant. Mycorrhizal inoculation enhanced the competitive ability of *V. faba*, with a greater aggressivity index and competitiveness than *B. napus*. In contrast, N-addition had less impact on interspecific interactions than AMF symbionts. Previous findings by Veiga et al. (2013) and Zhang et al. (2012) demonstrated the benefits of AMF inoculation on mycorrhizal plants ability for interspecific competition with regard to biomass and nutrients. Another study has revealed that the AMF community might induce variable plant growth responses (Munkvold et al., 2004). Considering our study, in both harvests, *V. faba* is the stronger competitor than *B. napus* in terms of plant growth responses, which reveals that AMF inoculation contributes to reduce the growth rate in non-host plants (Jordan et al., 2000; Facelli et al., 2010). Mycorrhizal inoculation improved the competitive ability of *V. faba*, which is consistent with previous study (Daisog et al., 2011). In contrast to these findings, different competing plants also indicated a decreasing trend in biomass in the presence of AMF than the NAMF treatments (Van Der Heijden et al., 2006). In the absence of competition or companionship, AMF generally reduces the growth performance of non-host plants (Muthukumar et al., 1997; Daisog et al., 2011), while it enhances the growth rate of host plant species (Ayres et al., 2006); however, exceptions are always there. For instance, previous findings suggested that both N-addition and inoculum from non-mycorrhizal plants reduced the AMF functions and growth of the mycorrhizal plant (De Souza and Santos, 2018). Mycorrhizal inoculation of non-host plant roots might develop into parasitic associations by altering plant defense mechanisms (Allen et al., 1989; Giovannetti and Lioi, 1990). Therefore, the AMF colonization was presumed in non-host plants, as detected in numerous species of Brassicaceae (Hirrel et al., 1978; Veiga et al., 2013), should ideally be categorized based on AMF to value the importance of these plant-AMF interactions. However, the underlying mechanism of such a phenomenon is still unknown.

In our study, in both harvests, maximum AMF colonization was reported in *V. faba* in the presence of high concentration of N. The potential competitive association of host plants is not just directly impacted by AMF colonization (Daisog et al., 2011; Lin et al., 2015) but also indirectly by altering the AMF community structure linked with the roots (Zhang et al., 2014; Qiao et al., 2015). Besides the impact of AMF on competitive association of host plant, the addition of N would improve the ability of plant photosynthesis, increasing the amount of C contribution into the fungal network, therefore enhancing colonization, and boosting C-P trade advantages (Evans, 1989; Unger et al., 2016). Consistent with these findings, other studies reported a significant competitive shift toward improved grass performance with increasing N levels (Hacker et al., 2015; Grygierzec et al., 2020). Contrary to these findings, the infectivity ability of AMF has been extensively decreased in different natural ecosystems by high levels of nitrogen (Smith and Read, 2008; Diepen et al., 2010; Hodge and Storer, 2014; Chen et al., 2017). The reduction of AMF colonization in monocultures under N-addition supporting the Johnson (2010) report that stated the C consumptions by mycorrhizal fungus. Apart from this, in the current study, N-addition unexpectedly had no effect on biomass of either plant species, signifying that N-addition does not offer any growth advantages.

Apart from above findings, *B. napus* showed negative responsiveness to AMF in mixed-culture, while stronger MGD was estimated for *V. faba* in monoculture at 2^nd^ harvest. In support of this, numerous prior works reported that AMF preferred legumes when competing against grass species (Klabi et al., 2014; Van Der Heijden et al., 2016; Bahadur et al., 2019b). AMF increase the availability of N and P to plants and improve the photosynthesis, ultimately leading to increased biomass (Yang et al., 2018; Begum et al., 2019; Vasan et al., 2021). Plant species with greater more mycorrhizal responsiveness generally have better nutrient uptake ability than less responsive species, which usually shows slow growth in the competition (Zabinski et al., 2002; Bahadur et al., 2019b). *Medicago sativa* showed high biomass production and broad canopy, which may deplete the biomass of nearby species, the below-ground drivers might be further exacerbated by aboveground competition (Klabi et al., 2014; Zhou et al., 2022). The involvement of AMF in plant nutrients uptake has been debated in different harvest periods with different N concentration, and the effect was varied from positive to negative (Schroeder-Moreno et al., 2012; Jiang et al., 2018). At high level of N concentration, the plant tissue N concentration was significantly enhanced by AMF inoculation, whereas it remained unaffected by a low level of N concentration in the greenhouse (Jackson et al., 2002; Yang et al., 2018). Here our study, AMF inoculation likely induced the flexible plant growth response, extending from beneficial to detrimental interaction. The MGDs ranged from highly positive to neutral, showing different contributions of AMF inoculation to plant growth. Our results recommended that the low P uptake by *B. napus* in N-addition might facilitate *V. faba*. Supporting our findings, previous studies showed that the growth of the host plant depends on the competitive pattern, AMF species, and nutrient availability (Hodge, 2003; Daisog et al., 2011). Therefore, the AMF community significantly contributes to plant growth performance and tissue N and P contents (both host and non-host plant) compared to a single AMF species.

Unlike previous studies (Höpfner et al., 2015; Bahadur et al., 2019b; Zhou et al., 2022), in this study the effect of competition on the AMF and N-addition were weak. In the current study, the inoculum contained the community of AMF, not the single-species of AMF. However, earlier research has confirmed that the mixed AMF inoculum treatment is more beneficial to the host than a single-species inoculum (Boyer et al., 2015; Armada et al., 2016). Due to the differences in growth strategies and nutrient acquisition by the competing plant species, it is hard to unravel the mechanisms by mycorrhizal fungi, which might alter plant performance in interspecific competition (Smith and Read, 2008; Unger et al., 2021). Recently, the specific mechanisms related to AMF colonization and its effects on the host and non-host plants to compete with N-addition have not been well approached. Explanations from this study point out that N-addition might alter the mycorrhizal colonization of the companion and host plants. Numerous studies have stated that the suppression of AMF colonization might impact the growth of host plants, leading to alteration in plant interactions (Qiao et al., 2016). In line with these findings, many studies have showed that higher competitive ability is associated with more excellent mycorrhizal growth responses of interspecific competitive interaction (Jansa et al., 2008; Johnson, 2010; Unger et al., 2021), consequently the simultaneous responses by N-addition and AMF and their interactions can affect plant nutrient uptake and acquisition.

## 5 Conclusion

This study reports the AMF community inoculation and N-addition patterns in quantified the competitive interactions in *V. faba* and B. napus. It was observed that AMF inoculation positively influenced the interspecific competitive ability of its host plant *V. faba*. Also, from the results it is evident that AMF might substantially impact the competitive interactions, growth performance, and tissue nutrient uptake between the host and non-host plant species. Furthermore, competitive interaction status depends on the presence of N-addition levels. However, *V. faba* increased shoot and root biomass when in mixed-culture, whereas AMF inoculation increased the N:P ratio in B. napus in mixed-culture under both N-addition treatments than NAMF plants at the 1^st^ harvest. Notably, little effects of N-addition on colonization growth were detected regardless of both harvests. In general, our results showed that N-addition reduced mycorrhizal growth dependency of B. napus, potentially weakening non-host competitiveness in N-addition treatments. Collectively, these findings provide information on how AMF functions in the competitive interaction in the absence of mycotrophic host plants with N-enrichments. To further increase understanding of plant competitive interactions under environmental change conditions, more research is required to determine how N deposition affects the function of AMF.

## Data availability statement

The raw data supporting the conclusions of this article will be made available by the authors, without undue reservation.

## Author contributions

AB, SJ, and JP conceived and designed the research. HF, YL, and QZ supervised the study. AB, SJ, MU, and JP performed greenhouse experiments. AB performed the microscopic study. AB, WS, and FN analyzed the data. WZ and MZ assisted with data valuation. AB, MU, and FN wrote the manuscript. TC and HF edited the manuscript writing. All authors contributed to the article and approved the submitted version.
