# Supplementary material for: Competitive interactions in two different plant species: Do grassland mycorrhizal communities and nitrogen addition play the same game?
Source: Front Plant Sci. 2023 Mar 13;14:1084218. doi: 10.3389/fpls.2023.1084218 (PMC10040756; doi:10.3389/fpls.2023.1084218)
Supplement: Supplementary file 1 [file Table_1.docx]

|  |  | **Total biomass** | | | |  | **Tissue N content** | | | |  | **Tissue P content** | | | |
| --- | --- | --- | --- | --- | --- | --- | --- | --- | --- | --- | --- | --- | --- | --- | --- |
|  |  | **MV** | **MCV** | **MB** | **MCB** |  | **MV** | **MCV** | **MB** | **MCB** |  | **MV** | **MCV** | **MB** | **MCB** |
| **1^st^ harvest** |  |  |  |  |  |  |  |  |  |  |  |  |  |  |  |
| NAMF |  |  |  |  |  |  |  |  |  |  |  |  |  |  |  |
| N-0 |  | 12.78±0.49d | 7.27±7.27b | 0.60±0.18a | 0.29±0.07a |  | 0.456±0.029c | 0.254±0.022b | 0.014±0.004a | 0.008±0.003a |  | 5.78±0.61d | 3.29±0.23b | 0.16±0.06a | 0.09±0.02a |
| N-15 |  | 13.44±0.45d | 8.72±0.78c | 1.31±0.19a | 0.27±0.03a |  | 0.458±0.051c | 0.295±0.041b | 0.033±0.006a | 0.008±0.003a |  | 6.39±0.52d | 4.53±0.60c | 0.40±0.07a | 0.08±0.02a |
| AMF |  |  |  |  |  |  |  |  |  |  |  |  |  |  |  |
| N-0 |  | 11.97±0.70c | 7.78±0.66b | 0.65±0.06a | 0.17±0.04a |  | 0.489±0.084c | 0.259±0.024b | 0.022±0.003a | 0.006±0.001a |  | 5.55±0.64c | 3.57±0.48b | 0.21±0.02a | 0.06±0.01a |
| N-15 |  | 11.98±0.23c | 8.92±1.13b | 0.33±0.05a | 0.14±0.06a |  | 0.457±0.034b | 0.284±0.046b | 0.010±0.002a | 0.005±0.002a |  | 4.25±0.35bc | 4.20±0.91bc | 0.10±0.02a | 0.05±0.02a |
| **2^nd^ harvest** |  |  |  |  |  |  |  |  |  |  |  |  |  |  |  |
| NAMF |  |  |  |  |  |  |  |  |  |  |  |  |  |  |  |
| N-0 |  | 14.55±0.32e | 8.81±0.29c | 1.20±0.19a | 1.14±0.10a |  | 0.986±0.016e | 0.723±0.056d | 0.072±0.011a | 0.079±0.005a |  | 11.82±1.05d | 7.64±0.38c | 0.49±0.12a | 0.47±0.04a |
| N-15 |  | 15.64±0.23f | 10.88±0.34d | 3.42±0.32b | 0.46±0.09a |  | 0.755±0.030d | 0.560±0.093c | 0.237±0.034b | 0.026±0.005a |  | 11.92±0.38d | 10.83±0.82d | 2.08±0.24b | 0.18±0.05a |
| AMF |  |  |  |  |  |  |  |  |  |  |  |  |  |  |  |
| N-0 |  | 18.41±0.60d | 11.83±0.64c | 2.27±0.21b | 0.45±0.07a |  | 1.063±0.064e | 0.904±0.050d | 0.177±0.014b | 0.035±0.007a |  | 11.34±0.55d | 8.24±0.38c | 1.31±0.22b | 0.23±0.05a |
| N-15 |  | 19.33±0.32d | 11.34±0.49c | 1.15±0.24ab | 0.33±0.06a |  | 1.273±0.064f | 0.784±0.022c | 0.030±0.006a | 0.018±0.003a |  | 12.92±0.19e | 8.24±0.27c | 0.39±0.08a | 0.12±0.03a |

**Table S1** In monoculture and mixed-culture *V. faba* and *B. napus* under AMF inoculation and N-addition conditions of N-0 and N-15. Means with the same letter in each column are not significantly different (Duncan test: *P* < 0.05). For further statistical details, see Table 2.

MV = Monoculture *V. faba*; MCV = Mixed-culture *V. faba*; MB = Monoculture *B. napus*; MCB = Mixed-culture *B. napus*
